# Supplementary material for: Ten-year cardiovascular risk among cancer survivors: The National Health and Nutrition Examination Survey
Source: PLoS One. 2021 Mar 4;16(3):e0247919. doi: 10.1371/journal.pone.0247919 (PMC7932508; doi:10.1371/journal.pone.0247919)
Supplement: S1 File — (DOCX) [file pone.0247919.s001.docx]

**S1 File**. Sensitivity analyses that include individuals with a history of CVD.

**S1 Table.** Estimated 10-year ASCVD risk

| ***Estimated ASCVD Risk*** | Total  N=17,291^1^ | | With a history of CVD  n=2,196^1^ | |
| --- | --- | --- | --- | --- |
| Pooled Cohort Equation (PCE) | mean±sd | 95% CI | mean±sd | 95% CI |
|  | 6.22±0.11 | 6.01, 6.44 | 12.95±0.34 | 12.27, 13.64 |
| Elevated 10-yr ASCVD Risk^2^ | n^1^ | % | n^1^ | % |
| No | 13,295 | 90.48% | 1,243 | 72.68% |
| Yes | 1,892 | 9.52% | 649 | 27.32% |

^1^ Unweighted sample size, n (%). All other analyses incorporated the NHANES sample weights

^2^ Elevated 10-year ASCVD risk was defined as a PCE ≥7.5%

**S2 Table.** Odds ratios (ORs) and 95% confidence intervals (CIs) for associations between cancer status and elevated vs. low 10-year ASCVD risk based on Pooled Cohort Equation, 2007-2016 NHANES

|  | | Elevated 10-year ASCVD Risk | | |
| --- | --- | --- | --- | --- |
|  |  | OR | 95% CI | P value |
| **Unadjusted Model** | |  |  |  |
|  | Positive cancer history vs. no cancer history | 2.79 | 2.32, 3.35 | <0.001 |
| **Adjusted Model** | |  |  |  |
|  | Positive cancer history vs. no cancer history | 2.93 | 2.24, 3.85 | <0.001 |

All analyses incorporated the NHANES sample weights

Adjusted Model controlled for BMI, race, marital status, education level, income to poverty ratio, dietary intake, physical activity, and depression score

*P _age*cancer_ Interaction <0.001*

**S3 Table.** Odds ratios (ORs) and 95% confidence intervals (CIs) for associations between cancer status and elevated vs. low 10-year ASCVD risk based on Pooled Cohort Equation according to cancer site, 2007-2016 NHANES

| **Type of cancer** | **Unweighted sample size, n=17,291^1^** | **weighted %** | **High 10-year CVD Risk** | | |
| --- | --- | --- | --- | --- | --- |
|  |  |  | OR | 95% CI | P value |
| No cancer history | 15,285 | 86.56% | reference | -- | -- |
| Breast^2^ | 332 | 1.93% | 1.86 | 0.94, 3.71 | 0.075 |
| **Bladder/Kidney** | 79 | 0.44% | 7.87 | 3.66, 16.97 | <0.001 |
| **Prostate^3^** | 307 | 1.22% | 6.23 | 3.17, 12.23 | <0.001 |
| **Colorectal** | 130 | 0.67% | 4.51 | 2.01, 10.13 | <0.001 |
| Other G/I | 47 | 0.18% | 3.37 | 0.98, 11.51 | 0.053 |
| Cervical^2^ | 109 | 0.89% | n/a | n/a | n/a |
| Ovary^2^ | 49 | 0.25% | 1.05 | 0.14, 7.51 | 0.964 |
| Uterus^2^ | 87 | 0.47% | n/a | n/a | n/a |
| Lung | 64 | 0.37% | 2.59 | 0.98, 6.88 | 0.055 |
| **Melanoma** | 114 | 1.05% | 3.87 | 1.88, 7.99 | <0.001 |
| Hematologic | 64 | 0.41% | 3.35 | 0.97, 11.57 | 0.056 |
| **Thyroid** | 44 | 0.31% | 0.11 | 0.01, 0.86 | 0.036 |
| **Testicular^3^** | 11 | 0.15% | 10.66 | 1.19, 95.72 | 0.035 |
| **Other** | 113 | 0.75% | 4.78 | 1.80, 12.67 | 0.002 |
| **Don't know** | 456 | 0.0435 | 3.24 | 1.94, 5.42 | <0.001 |

^1^ Unweighted sample size. All other analyses incorporated the NHANES sample weights

Adjusted Model controlled for BMI, race, marital status, education level, income to poverty ratio, dietary intake, physical activity, and depression score

Other G/I cancer included esophagus, gallbladder, liver, pancreas, and stomach; other cancer included bone, brain, nervous system, soft tissue, more than 3 cancers, and reported as other

^2^ with female participants as the comparison

^3^ with male participants as the comparison
