# Supplementary material for: Ten-year cardiovascular risk among cancer survivors: The National Health and Nutrition Examination Survey
Source: PLoS One. 2021 Mar 4;16(3):e0247919. doi: 10.1371/journal.pone.0247919 (PMC7932508; doi:10.1371/journal.pone.0247919)
Supplement: Attachment — Submitted filename: Response to Reviewers 2.15.docx [file pone.0247919.s003.docx]

Reviewer #2: The article has improved, and I have some minor comments to further improve the work.

The discussion section is much too long at 6 pages. Can the authors condense this?

We have significantly shortened the discussion as requested.

Figure 1 is helpful, but can the authors provide other figures of their findings? It is difficult to follow what is statistically significant and meaningful.

As requested, we added another figure (Figure 1) to graphically show multivariable-adjusted odds ratios for the association of cancer type and high 10-year CVD risk. To minimize the repetitive information, we removed table 4.

The number of abbreviations should be decreased. Clinicians are not familiar with terms like

PCEs.

Revised as requested. We kept a few abbreviations to make the article easy to follow.

The authors should discuss how their findings relate to pediatric cancer patients (in relationship to PMID 32298481), who typically have a long survival and are likely to die of competing causes. Also, the authors should relate their article on the recent SEER analysis on this topic: 32332714. Specifically, in these works, it appears that the likelihood death from heart disease increases after a cancer diagnosis; however, based on Figure 1, this does not seem to be the case. Can the authors better describe the findings from Figure 1? Can the authors discuss the relationship to the literature more?

Thank you for the comments. However, our paper focuses on the risk of 10-year high CVD risk, not the deaths from CVD. It is recognized cancer treatment increases the risk of CVD (PMID: 29337636, 31899651, 26919165, 30779651). Our work is based on 10-year CVD risk estimation, by cancer types and stratified by age groups. We are not able to compare our findings with research focus on CVD-specific deaths after cancer diagnosis.

I would remove this from the conclusions: “Adapting ASCVD risk assessment and acknowledging cardiovascular health following a cancer diagnosis is critical. Oncologists should advise patients with specific cancers of their potential high ASCVD risk and provide lifestyle modifications to reduce disease burden.” The authors may want to talk about this more in the discussion, but it is not what they found from the data.

We have removed this statement from the conclusions.
